# SGLT2 Inhibitors After Myocardial Infarction: Evidence, Mechanisms and Gaps in Knowledge

**DOI:** 10.3390/jcm15062260

**Published:** 2026-03-16

**Authors:** Angela Buonpane, Marco Ciardetti, Giancarlo Trimarchi, Giancarla Scalone, Michele Alessandro Coceani, Luigi Emilio Pastormerlo, Federica Marchi, Umberto Paradossi, Sergio Berti, Claudio Passino, Alberto Ranieri De Caterina

**Affiliations:** 1 Fondazione Toscana G. Monasterio, Ospedale del Cuore “G. Pasquinucci”, 54100 Massa, Italy; giancarlo.trimarchi@santannapisa.it (G.T.); scaloneg@ftgm.it (G.S.); lpastor@ftgm.it (L.E.P.); marchi@ftgm.it (F.M.); uparadossi@ftgm.it (U.P.); lfcberti@ftgm.it (S.B.); 2 Interdisciplinary Center for Health Sciences, Scuola Superiore Sant’Anna, 56127 Pisa, Italy; claudio.passino@santannapisa.it; 3 Fondazione Toscana G. Monasterio, Ospedale San Cataldo, 56124 Pisa, Italy; mciard@ftgm.it (M.C.); michecoc@ftgm.it (M.A.C.)

**Keywords:** myocardial infarction, heart failure, preventive cardiology, sodium–glucose co-transporter 2 inhibitors

## Abstract

Sodium–glucose cotransporter 2 inhibitors (SGLT2is) have revolutionized the treatment of heart failure and are now established as disease-modifying therapies across the spectrum of left ventricular ejection fraction. More recently, these agents have been evaluated in the early post-acute myocardial infarction (AMI) setting, raising interest in their potential role beyond heart failure prevention. Evidence from post-AMI randomized trials and contemporary meta-analyses consistently shows neutral effects on ischemic coronary outcomes, despite favorable effects on heart failure-related endpoints, ventricular remodeling, and cardiometabolic parameters. At the same time, data from experimental and translational research provide a biological framework in which SGLT2i exert anti-atherogenic effects through multiple complementary mechanisms, including improvement of cardiometabolic risk factors, attenuation of vascular and systemic inflammation, modulation of endothelial function, regulation of vascular smooth muscle cell behavior, macrophage inflammatory polarization, inhibition of inflammasome signaling, and modulation of the perivascular adipose tissue–vascular interface. Taken together, the available evidence highlights a dissociation between clinical trial outcomes in the early post-AMI phase and the underlying vascular biology associated with SGLT2 inhibition. While the dominant early clinical effects of SGLT2i appear to relate to hemodynamic and heart failure-preventive mechanisms, their potential impact on atherosclerotic disease may be more gradual and context-dependent. This review summarizes current clinical and mechanistic evidence supporting this interpretation and discusses the implications for understanding the role of SGLT2i in patients after AMI.

## 1. Introduction

### 1.1. “Absence of Evidence Is Not Evidence of Absence” (Carl Sagan, Astronomer)

Acute myocardial infarction (AMI) remains a major cause of heart failure (HF), recurrent cardiovascular events, and long-term morbidity despite substantial advances in reperfusion strategies and secondary prevention. While contemporary therapies have markedly improved early survival, the post-AMI phase continues to be characterized by a high residual risk of adverse ventricular remodeling, progression to HF, and recurrent ischemic events, underscoring the need for disease-modifying strategies capable of influencing long-term cardiovascular trajectories. Sodium–glucose cotransporter 2 inhibitors (SGLT2is) have emerged as a major therapeutic breakthrough in cardiovascular medicine. Initially developed as glucose-lowering agents, these drugs have demonstrated robust and consistent benefits in HF across the entire spectrum of left ventricular ejection fraction, independent of diabetes status, establishing them as foundational disease-modifying therapies [1]. In recent years, interest has grown in the potential role of SGLT2i in the early post-AMI setting. However, several trials initiated after AMI have not shown consistent reductions in recurrent ischemic coronary events, despite favorable effects on HF-related outcomes, ventricular remodeling, and cardiometabolic parameters [2,3,4,5,6,7,8]. At the same time, a growing body of experimental and translational evidence indicates that SGLT2i exert anti-inflammatory and anti-atherogenic effects, acting on key biological processes involved in atherogenesis and plaque vulnerability [9,10,11]. These observations introduce an apparent dissociation between neutral ischemic trial outcomes in the early post-AMI phase and compelling biological mechanisms that would be expected to favor vascular protection. The aim of this review is to summarize current evidence on the use of SGLT2 inhibitors after myocardial infarction and discuss the mechanistic basis underlying their cardiovascular effects. Particular attention is given to anti-inflammatory and anti-atherogenic pathways that may influence plaque biology and disease progression over time, providing a framework to interpret why the benefits of SGLT2i in the post-AMI setting appear to preferentially involve heart failure prevention rather than early reductions in ischemic events.

### 1.2. Methods

This narrative review was based on a structured literature search performed in PubMed/MEDLINE and Scopus up to February 2026. Search terms included combinations of “SGLT2 inhibitors”, “myocardial infarction”, “acute coronary syndrome”, “atherosclerosis”, “inflammation”, “heart failure”, “plaque”, and “vascular remodeling”. Randomized clinical trials, meta-analyses, mechanistic studies, and relevant review articles published in English were considered. Additional references were identified by screening the bibliographies of selected articles. Studies were selected on the basis of relevance to the topic and methodological quality, with particular attention to post-AMI clinical evidence and experimental data exploring vascular and anti-atherogenic mechanisms.

## 2. SGLT2i from Glucose Lowering to Cardiovascular Disease-Modifying Therapy

SGLT2i were initially developed as glucose-lowering agents for the treatment of type 2 diabetes mellitus (T2D); however, large cardiovascular outcome trials have demonstrated benefits that extend well beyond glycemic control. In patients with T2D, largely without symptomatic HF at baseline, empagliflozin in EMPA-REG OUTCOME [12] and canagliflozin in the CANVAS Program [13] significantly reduced major adverse cardiovascular events and heart failure hospitalizations, while dapagliflozin in DECLARE–TIMI 58 [14] reduced the composite of cardiovascular death or heart failure hospitalization, with the benefit predominantly driven by heart failure outcomes. Ertugliflozin in VERTIS-CV [15] showed neutral effects on atherosclerotic endpoints but consistently reduced heart failure hospitalizations, whereas sotagliflozin in SCORED [16] further supported a class effect on heart failure prevention in patients with diabetes. Collectively, these data established SGLT2i as effective agents for reducing the risk of heart failure and cardiovascular events in patients with T2D, particularly in those with established cardiovascular disease or high cardiovascular risk, forming the basis of current European Society of Cardiology (ESC) recommendations for glucose-lowering therapy with cardiovascular benefit [1].

In patients with established HF, irrespective of diabetes status, SGLT2i have emerged as foundational disease-modifying therapies. In HF with reduced ejection fraction (HFrEF), dapagliflozin in DAPA-HF [17] and empagliflozin in EMPEROR-Reduced [18] significantly reduced the risk of the respective primary composites (worsening HF or cardiovascular death in DAPA-HF; cardiovascular death or HF hospitalization in EMPEROR-Reduced), with consistent benefits in patients with and without T2D; additionally, the SGLT1/2i sotagliflozin reduced cardiovascular death and HF events in patients with T2D and recent worsening HF in SOLOIST-WHF [19], supporting the strong positioning of SGLT2 inhibition in the 2021 ESC Heart Failure Guidelines [1]. More recently, evidence has expanded to patients with mildly reduced (HFmEF) and preserved ejection fraction (HFpEF). In EMPEROR-Preserved [20] and DELIVER [21], empagliflozin and dapagliflozin, respectively, reduced the composite of cardiovascular death or worsening HF in patients with LVEF >40%, with benefits largely driven by reductions in HF hospitalizations that were consistent irrespective of diabetes status; accordingly, the 2023 ESC Focused Update [22] recommends dapagliflozin or empagliflozin in HFmrEF and HFpEF (Class I, Level A) to reduce the risk of HF hospitalization or cardiovascular death, while noting that the observed effect in these trials was predominantly due to fewer HF hospitalizations rather than reduced cardiovascular mortality.

### 2.1. Mechanisms Underlying the Beneficial Effects of SGLT2 Inhibitors in HF

Accumulating evidence indicates that the cardiovascular benefits of SGLT2i in HF derive from a combination of renal, hemodynamic, metabolic, and myocardial mechanisms that extend beyond glycemic control. No single pathway fully explains the magnitude and consistency of benefit observed across HF phenotypes; rather, SGLT2 inhibition appears to act as an integrated cardio–renal–metabolic intervention targeting multiple pathophysiological processes simultaneously.

#### 2.1.1. Hemodynamic and Renal Effects

By inhibiting sodium and glucose reabsorption in the proximal renal tubule, SGLT2i induce natriuresis and osmotic diuresis, leading to modest reductions in blood pressure and plasma volume [9]. Importantly, experimental and clinical data suggest that this volume reduction preferentially affects the interstitial compartment rather than the intravascular space, thereby alleviating tissue congestion while minimizing reductions in effective arterial blood volume [9]. This distinctive pattern of decongestion attenuates cardiac preload and ventricular wall stress without triggering excessive sympathetic or renin–angiotensin–aldosterone system activation, mechanistically distinguishing SGLT2i from conventional loop or thiazide diuretics [23,24]. In parallel, the increased delivery of sodium to the macula densa enhances tubuloglomerular feedback signaling, resulting in suppression of renin release from juxtaglomerular cells and a downstream attenuation of RAAS activity, an effect that resembles a RAAS inhibitor-like profile without direct pharmacological blockade [25].

These renal hemodynamic effects occur rapidly after treatment initiation and are thought to account, at least in part, for the early and consistent reduction in HF hospitalizations observed in large outcome trials [17,18,20]. Furthermore, improved tubuloglomerular feedback and reduced intraglomerular pressure may indirectly contribute to cardiovascular benefit by alleviating renal stress, dampening neurohumoral activation, and reducing systemic inflammation [9].

#### 2.1.2. Myocardial Energy Metabolism and Remodeling

HF is characterized by profound alterations in myocardial substrate utilization, mitochondrial dysfunction, and a progressive decline in adenosine triphosphate (ATP) availability [26]. SGLT2i induce a systemic metabolic shift toward increased lipolysis and hepatic ketogenesis, resulting in elevated circulating ketone bodies—particularly β-hydroxybutyrate—which are readily taken up and oxidized by the failing myocardium [27]. Rather than acting as a more energy-efficient substrate per se, ketones appear to serve as an additional and complementary fuel source for the energy-starved heart. Experimental evidence indicates that SGLT2 inhibition increases total myocardial ATP production without suppressing glucose or fatty acid oxidation, thereby enhancing overall energy availability. This improvement in energetic reserve supports contractile performance, reduces cellular stress, and may contribute to attenuation of adverse ventricular remodeling [28]. Consistent with this concept, SGLT2i have been shown to reduce left ventricular mass, myocardial fibrosis, and hypertrophy in both preclinical models and human studies. These effects appear to be relevant across the entire spectrum of left ventricular ejection fraction, supporting the broad efficacy of SGLT2i in both HFrEF and HFpEF [9].

#### 2.1.3. Anti-Inflammatory and Anti-Fibrotic Effects

Chronic low-grade inflammation is a central driver of HF progression, contributing to endothelial dysfunction, extracellular matrix turnover, myocardial fibrosis, and adverse ventricular remodeling [29,30,31]. Elevated circulating inflammatory cytokines and activation of innate immune pathways correlate closely with HF severity and prognosis, irrespective of ejection fraction [32,33]. Emerging evidence strongly supports a potent anti-inflammatory role of SGLT2is, which may represent a key unifying mechanism underlying their cardiovascular benefits.

One of the most compelling mechanistic insights relates to inhibition of the NOD-like receptor family, the pyrin domain-containing 3 (NLRP3) inflammasome [34,35], a cytosolic multiprotein complex that plays a pivotal role in innate immune activation and has been directly implicated in HF pathophysiology [36]. Activation of the NLRP3 inflammasome promotes the maturation and release of interleukin-1β and interleukin-18, driving myocardial inflammation, fibrosis, and contractile dysfunction [36]. SGLT2i have been shown to suppress NLRP3 inflammasome activation across multiple tissues, including the heart, kidney, vasculature, liver, and immune cells, through mechanisms that appear to be at least partially independent of glucose lowering [9,32,34,35]. Notably, increased circulating levels of β-hydroxybutyrate induced by SGLT2i may directly inhibit NLRP3 inflammasome activation, providing a mechanistic link between metabolic reprogramming and immunomodulation [37].

In parallel, SGLT2i reduce oxidative stress and reactive oxygen species (ROS) generation, which are known upstream activators of inflammatory cascades and fibrotic signaling [29,30]. Mitochondrial overproduction of ROS represents a central mechanism contributing to myocardial dysfunction and adverse remodeling in HF [30,38] in a vicious cycle in which progressive mitochondrial impairment in the failing heart is both a cause and a consequence of oxidative stress. In this context, increased ROS generation arises from maladaptive stimulation of mitochondrial respiration aimed at sustaining ATP production. Enhanced electron transport chain activity in dysfunctional mitochondria favors electron leak and oxidative damage, which further disrupt mitochondrial integrity and activate pro-fibrotic and pro-inflammatory signaling pathways [38]. SGLT2 inhibition has been shown to mitigate this oxidative burden in experimental settings. In diabetic mouse models, SGLT2 inhibitors reduce myocardial ROS levels and attenuate cardiac fibrosis, indicating a link between improved metabolic control and reduced oxidative injury [39].

Taken together, these complementary mechanisms explain the early onset, durability, and diabetes-independent benefits of SGLT2 inhibitors in heart failure. The integrated nature of their actions supports their classification as disease-modifying cardio–renal therapies rather than conventional glucose-lowering agents and underpins their central role in contemporary heart failure management.

## 3. Role of SGLT2i in Acute Coronary Syndromes: Current Evidence and Guideline Positioning

### 3.1. Evidence from Randomized Trials

In parallel with the expanding mechanistic rationale supporting SGLT2i as cardio–renal–metabolic modulators, considerable attention has been directed toward their potential role in the early post-AMI setting; however, the body of randomized evidence generated to date has consistently failed to demonstrate a reduction in major adverse cardiovascular events (MACEs) when these are defined as recurrent ischemic coronary outcomes (Table 1). Early small randomized studies exploring empagliflozin after acute coronary syndromes (ACS), such as the trial by Adel et al. [2], did not show reductions in cardiovascular death, recurrent myocardial infarction, stroke, or repeat revascularization, highlighting from the outset the absence of a signal on ischemic events despite acceptable safety. Subsequent mechanistic trials shifted focus toward surrogate endpoints: EMBODY [3] demonstrated improvements in cardiac autonomic balance following empagliflozin initiation after MI, suggesting potential anti-arrhythmic or neurohumoral effects. This paradigm was further supported by EMMY [4], in which early empagliflozin initiation within days of reperfused MI resulted in greater reductions in NT-proBNP and improvements in left ventricular volumes and function over follow-up, yet the trial was explicitly not powered to assess mortality or ischemic events. Comparable findings emerged with dapagliflozin in DACAMI [5], conducted in non-diabetic patients with anterior ST-elevation myocardial infarction (STEMI), where modest improvements in biomarkers and ventricular remodelling were observed. Importantly, similar to the aforementioned mechanistic studies, DACAMI was not designed nor powered to evaluate recurrent ischemic endpoints such as myocardial infarction, stroke, or coronary revascularization. Even very early administration strategies failed to modify coronary endpoints: in EMI-STEMI [6], empagliflozin given before primary PCI did not reduce infarct size or inflammatory markers, despite a small increase in left ventricular ejection fraction at short-term follow-up. Larger outcome-oriented trials confirmed this pattern. In DAPA-MI [7], conducted in patients without T2D or chronic HF, dapagliflozin did not reduce cardiovascular death, recurrent MI, or HF hospitalization, leading to a shift toward a hierarchical cardiometabolic composite endpoint that was driven by non-ischemic components. Most definitively, EMPACT-MI [8], the largest event-driven trial in this setting, failed to reduce its primary composite of all-cause death or HF hospitalization, although a reduction in first HF hospitalization was observed; critically, no benefit was seen for mortality or recurrent coronary events. Taken together, these trials consistently demonstrate that while SGLT2i initiated after AMI exert favorable effects on biomarkers, ventricular remodeling, autonomic tone, and HF-related outcomes, they do not modify the incidence of ischemic MACEs, underscoring a mechanistic profile that is predominantly hemodynamic and myocardial rather than anti-atherothrombotic. This interpretation is further reinforced by multiple contemporary meta-analyses and systematic reviews that uniformly show no reduction in MACEs, cardiovascular or all-cause mortality, recurrent myocardial infarction, or stroke with early SGLT2i initiation, while consistently demonstrating a significant reduction in HF events and modest improvements in left ventricular ejection fraction [40,41,42,43,44,45].

### 3.2. Interpretation of Trial Results

Overall, randomized trials evaluating SGLT2i in the early post-AMI setting have not demonstrated significant reductions in recurrent ischemic events. However, several studies have suggested favorable effects on cardiac remodeling and HF-related outcomes, including improvements in ventricular function and reductions in HF-related events. These findings suggest that the early benefits of SGLT2 inhibition after AMI may be driven predominantly by hemodynamic and metabolic mechanisms influencing ventricular remodeling rather than by immediate modification of coronary atherosclerotic processes. Several factors may explain the neutral effect on recurrent ischemic endpoints observed in post-AMI trials evaluating SGLT2is. First, most studies had relatively short follow-up durations, which may be insufficient to capture potential effects on atherosclerotic plaque biology. Second, contemporary post-AMI populations receive intensive background therapy, including early revascularization, dual antiplatelet therapy, high-intensity lipid-lowering treatment, and optimized secondary prevention, which substantially lowers the residual risk of recurrent ischemic events. Third, the early benefits of SGLT2i after AMI may be predominantly mediated by hemodynamic and metabolic effects influencing ventricular remodeling and HF risk rather than by immediate modification of coronary atherosclerotic processes. Finally, potential anti-atherogenic effects may require longer exposure or may become clinically relevant only in selected high-risk phenotypes characterized by persistent inflammatory or metabolic risk.

### 3.3. Safety Considerations in the Early Post-Myocardial Infarction Setting

Although SGLT2i have demonstrated a favorable safety profile in large cardiovascular outcome trials, specific considerations apply when initiating therapy in the early post-AMI setting. In patients with ACS, potential concerns include transient reductions in estimated glomerular filtration rate, volume depletion or hypotension in hemodynamically unstable individuals, and the rare occurrence of euglycemic ketoacidosis, particularly in patients with diabetes. Careful patient selection and monitoring of renal function, volume status, and metabolic parameters are therefore recommended when considering SGLT2i initiation during the early post-infarction phase. In addition, potential interactions with contemporary ACS pharmacotherapy—including diuretics, renin–angiotensin system inhibitors, and other cardioprotective agents—should be taken into account to minimize the risk of hypotension or renal dysfunction. Overall, available randomized trials conducted in post-AMI populations have reported acceptable safety profiles, although most studies excluded patients with severe hemodynamic instability, highlighting the need for cautious use and further evidence in higher-risk clinical scenarios.

### 3.4. Current Guideline Positioning

In light of the available evidence, current ESC guidelines on ACS recommend SGLT2i in the context of optimal glucose-lowering therapy for patients with T2D, but not as routine post-infarction cardiovascular treatment [46]. Despite their established benefits in T2D and HF, clinical trials evaluating early initiation of SGLT2i after AMI have not demonstrated consistent reductions in ischemic cardiovascular events. Accordingly, SGLT2i are not currently recommended solely on the basis of an ACS diagnosis in patients without HF or T2D, and their potential role in this setting remains to be defined by dedicated outcome-driven randomized trials.

**Table 1:** Main characteristics of studies evaluating SGLT2i in patients after acute myocardial infarction.

Study ID	Sample Size SGLT2i/Control (N)	Dose and Timing of SGLT2i	FU Duration	Endpoint	Results
Adel et al. 2022 [2]	45/48	Empagliflozin, 10 mg once daily, immediately after PCI.	6 months	Primary: composite of all-cause mortality, coronary revascularization, rehospitalization due to UAP, hospitalization due to HF, cardiovascular death, non-fatal MI, and non-fatal stroke.	No differences in MACEs between the two groups.
Shimizu et al. 2020 (EMBODY) [3]	46/50	Empagliflozin, 10 mg once daily, 2 weeks after the onset of AMI.	6 months	Primary: the change from baseline to 24 weeks in heart rate variability.	Improved autonomic markers in experimental group.
Lewinski et al. 2022 (EMMY) [4]	237/239	Empagliflozin, 10 mg once daily, 72 h after PCI.	6 months	Primary endpoint: changes in NT-proBNP at 6 months. Secondary: changes in NT-proBNP at 6 weeks, changes in LVEF at 6 and 26 weeks, changes in LVESV and LVEDV, and changes in ketone body, glycated hemoglobin concentrations, and body weight.	Greater NT-proBNP reduction over 26 weeks and significant improvement in echocardiographic functional and structural parameters in experimental group.
Dayem et al. 2023 (DACAMI) [5]	50/50	Dapagliflozin, 10 mg once daily, 72 h post-anterior STEMI.	3 months	Primary: NT-proBNP change over 12 weeks post-anterior STEMI and/or a change in LVEF, EDV, and/or LVMi.	Significant NT-proBNP reduction and change in LVMi in the experimental group.
Khani et al. 2024 (EMI-STEMI) [6]	53/53	Empagliflozin, 10 mg once daily, immediately before PCI.	6 months	Primary: cTnI change, estimated AUC and peak of cTnI, resolution of ST-segment in >50% of leads 90 min after PCI, and changes in LVEF 40 days after primary PCI. Secondary: hs-CRP changes at discharge, TIMI flow after PCI, and the occurrence of MACEs, including hospitalization, re-MI, stroke, and death.	No difference in infarct size markers or ST resolution between the two groups; significant increase in LVEF in the experimental group; no difference in MACEs.
James et al. 2023 (DAPA-MI) [7]	2019/1998	Dapagliflozin, 10 mg once daily, during hospitalization for the index MI event or within 10 days from index MI.	12 ± 1 months	Primary: hierarchical composite of death, HF hospitalization, nonfatal MI, AF/flutter, new T2D, NYHA class, >5% weight loss. Key secondary: hierarchical composite (excluding weight). Other Secondary and Exploratory End Points: composite of CV death/hospitalization for HF, MACEs (MI, stroke, or CV death), all-cause death, CV death, MI, and stroke.	Significant benefit driven by cardiometabolic components adjudicated HF hospitalization, new T2D, NYHA class, >5% weight loss. No differences in MACEs (MI, stroke, or CV death), all-cause death, CV death, stroke, or MI.
Butler et al. 2024 (EMPACT-MI) [8]	3260/3262	Empagliflozin, 10 mg once daily, within 14 days after admission.	17.9 months	Primary: composite of hospitalization for HF or death from any cause. Secondary: HF hospitalizations or death from any cause, nonelective cardiovascular hospitalizations or death from any cause, non-elective hospitalizations for any cause or death from any cause, and hospitalizations for MI or death from any cause.	No differences between the two groups.

PCI: percutaneous coronary intervention; UAP: unstable angina pectoris; HF: heart failure; MI: myocardial infarction; MACEs: major adverse cardiovascular events; AMI: acute myocardial infarction; NT-proBNP: N-terminal pro-hormone of brain natriuretic peptide; LVEDV: left ventricular end-diastolic volume; LVESV: left ventricular end-systolic volume; LVEF: left ventricular ejection fraction; LVMi: left ventricular mass index; TIMI: thrombolysis in myocardial infarction; T2D: type 2 diabetes mellitus; NYHA: New York Heart Association; CV: cardiovascular.

## 4. Anti-Atherogenic Mechanisms of SGLT2 Inhibitors Beyond Ischemic Clinical Endpoints

While post-AMI randomized trials have not demonstrated reductions in recurrent ischemic coronary events, preclinical and translational studies suggest that SGLT2i may influence atherosclerotic biology through multiple vascular and inflammatory pathways. These mechanistic observations should be interpreted as biologically plausible but not yet clinically proven anti-atherogenic effects in the post-AMI setting.

Across both clinical and preclinical studies, SGLT2i have consistently been shown to reduce fasting blood glucose, hemoglobin A1c, body weight, total and low-density lipoprotein (LDL) cholesterol, triglycerides, and blood pressure, factors that are well established as key drivers of atherogenesis [47]. Through this broad and convergent improvement of cardiometabolic risk factors, SGLT2i may exert anti-atherogenic effects, largely mediated by improvements in cardiometabolic risk factors that contribute to endothelial dysfunction, vascular inflammation, and plaque progression. Moreover, beyond this risk factor-mediated effect, a coherent mechanistic framework supports the concept that these drugs may act upstream of clinical events, attenuating key biological drivers of atherogenesis and plaque vulnerability. Such effects appear to be mediated both indirectly, through systemic metabolic and anti-inflammatory actions, and directly, through modulation of cellular pathways operating within the vascular wall. In this context, endothelial cells, vascular smooth muscle cells (VSMCs), macrophages, and the perivascular adipose tissue (PVAT)–vascular interface emerge as relevant biological targets through which SGLT2i may influence plaque biology without necessarily translating into short-term reductions in ischemic events [9,10,48].

### 4.1. Inflammation and Oxidative Stress as Central Drivers of Atherogenesis

Chronic low-grade inflammation represents a central driver of atherosclerosis, linking metabolic stress to endothelial dysfunction and plaque progression [49]. As discussed in the section on heart failure mechanisms, SGLT2 inhibitors have been shown in experimental and translational studies to modulate several inflammatory pathways, including NF-κB signaling and NLRP3 inflammasome activation. These mechanisms, initially described in cardiometabolic and heart failure models, may also be relevant to vascular inflammation and atherosclerotic plaque biology. Experimental studies suggest that SGLT2 inhibition may attenuate vascular inflammatory signaling, with reductions in the expression of pro-inflammatory cytokines and adhesion molecules involved in leukocyte recruitment and vascular inflammation [50,51,52]. In addition, improvements in cellular metabolic homeostasis and attenuation of oxidative stress may contribute to limiting inflammatory responses within the vascular wall [53,54].

Within this framework, modulation of the NLRP3 inflammasome has been proposed as a potential mechanistic link between SGLT2 inhibition and plaque inflammation. Preclinical evidence suggests that SGLT2 inhibitors may reduce inflammasome activation through mechanisms related to improved cellular metabolism, enhanced autophagy, and reduced oxidative stress, with downstream attenuation of IL-1β-mediated inflammatory signaling [9,32,34,35]. Such modulation supports a shift toward a less inflammatory plaque milieu, which may theoretically influence plaque biology, although direct evidence of plaque stabilization in humans remains limited. However, these observations derive predominantly from experimental models, and direct confirmation of inflammasome-mediated vascular effects in humans remains limited.

### 4.2. Endothelial Cells: Preservation of Vascular Homeostasis

Endothelial dysfunction is a pivotal initiating event in atherogenesis, particularly in diabetes and metabolic disease. Preclinical evidence indicates that SGLT2i improve endothelial function by enhancing endothelial nitric oxide synthase (eNOS) phosphorylation, increasing nitric oxide bioavailability, and reducing endothelial activation [55,56,57]. These effects are accompanied by downregulation of adhesion molecules and inflammatory mediators, thereby limiting leukocyte adhesion and transmigration into the arterial wall [50,51,52].

In addition to functional improvements, SGLT2 inhibition appears to preserve endothelial structural integrity by mitigating oxidative stress-induced damage, preventing eNOS uncoupling, and improving endothelial glycocalyx integrity in experimental models. Improvements in arterial stiffness and microvascular function reported in preclinical studies further support the concept that SGLT2i act upstream in the atherosclerotic cascade by restoring endothelial homeostatic mechanisms [9,10].

### 4.3. Vascular Smooth Muscle Cells: Modulation of Phenotypic Switching and Plaque Architecture

VSMCs play a critical role in both plaque progression and stabilization. Under metabolic and inflammatory stress, VSMCs undergo phenotypic switching toward a proliferative, migratory, and pro-inflammatory state, contributing to intimal thickening and plaque growth [58,59]. Experimental evidence suggests that SGLT2i attenuate these maladaptive responses by reducing oxidative stress and inflammatory signaling within the vascular wall [11,33,60]. Importantly, NLRP3 inflammasome activation has been described not only in macrophages but also in VSMCs, where it contributes to plaque inflammation and necrotic core expansion [60]. By dampening inflammasome-related signaling, SGLT2i may indirectly favor a more stable plaque phenotype characterized by preserved VSMC content and reduced inflammatory degradation of the fibrous cap, thereby influencing plaque architecture rather than plaque size alone.

### 4.4. Macrophage Biology and Inflammatory Polarization

Macrophages are central orchestrators of atherosclerotic inflammation, driving foam cell formation, cytokine release, and necrotic core development. Multiple preclinical studies indicate that SGLT2 inhibitors reduce macrophage recruitment into the vessel wall and attenuate macrophage-driven inflammatory signaling. Beyond limiting macrophage burden, SGLT2 inhibition has been associated with a functional shift in macrophage phenotype, favoring polarization toward a more anti-inflammatory, M2-like profile, alongside reduced secretion of IL-1β, IL-18, and TNF-α [61,62].

This phenotypic modulation is mechanistically linked to suppression of NF-κB and NLRP3 inflammasome activation [9,32,34,35], as well as restoration of macrophage autophagy [63,64], which limits cholesterol crystal-induced inflammasome hyperactivation. Collectively, these effects are consistent with mechanisms that may influence plaque inflammation and stability, although these observations derive primarily from experimental models.

### 4.5. Perivascular Adipose Tissue: Modulation of the Adipose–Vascular Inflammatory Axis

PVAT is no longer considered a passive structural support of the vascular wall but rather a dynamic metabolic and inflammatory compartment that actively participates in vascular homeostasis and disease [65]. PVAT surrounds the coronary arteries and is uniquely positioned to engage in a bidirectional cross-talk with the underlying vessel wall. Through the release of adipokines, cytokines, and vasoactive mediators, PVAT influences endothelial function, vascular tone, and inflammatory signaling. Conversely, inflammatory and oxidative signals originating from the arterial wall modulate PVAT phenotype and secretory activity, creating a reciprocal interaction in which vascular inflammation and PVAT dysfunction reinforce each other [66,67].

In cardiometabolic disease, this tightly regulated cross-talk becomes maladaptive. PVAT undergoes qualitative and quantitative remodeling characterized by adipocyte hypertrophy, increased lipid accumulation, immune cell infiltration, and enhanced production of pro-inflammatory and profibrotic mediators [67]. This dysfunctional PVAT phenotype amplifies local vascular inflammation, promotes endothelial dysfunction and arterial stiffening, and sustains a pro-atherogenic microenvironment around the coronary arteries. At the same time, inflamed coronary segments further exacerbate PVAT dysfunction, establishing a feed-forward loop in which PVAT inflammation mirrors and propagates coronary inflammation.

Importantly, the inflammatory state of PVAT can be non-invasively captured and quantified using cardiac computed tomography angiography (CCTA) [68,69]. Changes in PVAT composition can be quantified through the fat attenuation index (FAI), which reflects alterations in adipocyte lipid–water content associated with coronary inflammation [67,68,70]. In this sense, PVAT inflammation serves as a functional “thermometer” of coronary inflammatory activity, providing dynamic information on disease activity that extends beyond static measures of plaque burden or luminal stenosis [66,67]. CCTA studies have demonstrated that elevated perivascular FAI is a strong predictor of cardiac mortality and major adverse cardiovascular events, independent of traditional risk factors and plaque burden [71].

Comparative studies integrating intravascular imaging modalities with CCTA further reinforce the pathophysiological relevance of PVAT. Increased PVAT attenuation on CCTA has been associated with optical coherence tomography features of plaque vulnerability, including lipid-rich plaques, macrophage infiltration, plaque rupture, and microchannel [72,73]. Taken together, these imaging data strongly support the concept that PVAT is not merely associated with atherosclerosis but actively reflects and amplifies local vascular inflammation and plaque vulnerability.

Within this context, SGLT2i exert biologically relevant effects on PVAT by reducing both adipose tissue mass and inflammatory activation. SGLT2 inhibition is associated with attenuation of perivascular and epicardial adipose tissue accumulation [74], together with a reduction in the local release of bioactive molecules such as leptin, TNF-α, and plasminogen activator inhibitor-1 [75]. These changes limit the paracrine pro-inflammatory and profibrotic actions of PVAT on the coronary vasculature and myocardium, contributing to a more favorable vascular and cardiac microenvironment.

Through these mechanisms, modulation of PVAT may represent an additional pathway through which SGLT2i influence vascular inflammation and plaque biology. However, these observations remain largely derived from experimental and translational evidence, and direct clinical confirmation in humans is still limited.

## 5. How to Reconcile “Anti-Atherogenic Biology” with Neutral Post-AMI Ischemic MACE Trials

SGLT2 inhibitors exert pleiotropic biological effects that plausibly intersect with atherosclerotic disease, including endothelial protection, immune and inflammatory modulation, and broad metabolic and lipid-related improvements. However, the lack of consistent reductions in coronary ischemic endpoints can be mechanistically explained by the nature, timing, and context of these effects. The vascular actions of SGLT2 inhibition appear to operate predominantly upstream of acute ischemic events, with early benefits largely driven by hemodynamic unloading, attenuation of adverse ventricular remodeling, and prevention of heart failure progression. In contrast, anti-atherogenic effects are likely to develop more slowly, be strongly dependent on metabolic phenotype, and be incremental when superimposed on contemporary background therapy with potent antithrombotic and lipid-lowering agents. Within this framework, neutral findings for coronary MACEs in the early post-AMI phase, as evidenced by contemporary clinical trials, are biologically expected rather than paradoxical, as relatively short follow-up durations and low event rates in modern MI populations may be insufficiently sensitive to capture gradual, phenotype-dependent modifications in plaque biology.

If SGLT2i truly have anti-atherogenic effects, the expected clinical signature would likely require (i) longer follow-up, (ii) higher baseline inflammatory/metabolic risk, and (iii) endpoints enriched for progressive atherosclerosis (or imaging of plaque vulnerability/inflammation) rather than early post-ACS recurrence dominated by plaque rupture, thrombosis, and the powerful effects of dual antiplatelet therapy (DAPT) or statins. In other words, asking SGLT2i to behave like a statin, colchicine, or Proprotein Convertase Subtilisin/Kexin Type 9 inhibitors (PCSK9is) in the early post-MI window may be the wrong test; their most coherent role is to bend the post-MI HF and remodeling curve early, while any anti-atherosclerotic effect—if clinically meaningful—may be a slower “residual risk” modifier best detected in longer-term disease-modification frameworks.

### Timing of Benefit After Acute Myocardial Infarction: Contrasting Antithrombotic, Lipid-Lowering, Anti-Inflammatory, and SGLT2-Inhibitor Therapies

After AMI, the temporal pattern with which different pharmacological strategies reduce clinical events reflects their dominant pathophysiological targets. Therapies that directly inhibit platelet aggregation and thrombosis, such as dual antiplatelet therapy and anticoagulation when indicated, exert their maximal benefit early after the index event, when recurrent ischemic risk is highest and largely driven by plaque rupture and thrombus propagation. In contrast, lipid-lowering therapies, including high-intensity statins and PCSK9is, primarily act by stabilizing atherosclerotic plaques and reducing lipid burden; although statins may exert early pleiotropic anti-inflammatory effects, consistent reductions in ischemic events typically emerge over months, in parallel with progressive plaque stabilization and regression.

Anti-inflammatory therapies provide an instructive comparator for understanding the time course of disease-modifying effects on atherosclerosis. In trials targeting residual inflammatory risk, benefits on ischemic outcomes were not immediate but accrued gradually. In CANTOS, canakinumab reduced recurrent cardiovascular events only after sufficient follow-up (48 months), consistent with the concept that suppression of interleukin-1β-driven inflammation modifies plaque biology and vulnerability over time [76]. Similarly, colchicine trials have demonstrated a temporal pattern aligned with progressive anti-inflammatory plaque stabilization. In COLCOT [77], initiated early after myocardial infarction, separation of event curves emerged over subsequent months (median follow-up 22.6 months) rather than in the immediate post-AMI phase, while in LoDoCo2 [78], a study conducted on stable chronic coronary disease, colchicine reduced ischemic events over longer-term follow-up (median follow-up 28.6 months). These observations reinforce a key principle: anti-inflammatory strategies reduce atherothrombotic events by altering plaque inflammation and stability, a process that requires time to translate into fewer clinical events, even when initiated early after ACS.

SGLT2i follow a distinct but partially overlapping temporal trajectory. Their earliest and most reproducible benefits arise from hemodynamic and cardio–renal mechanisms, which explain the rapid and consistent reduction in HF hospitalizations observed across multiple clinical settings. By contrast, any potential anti-atherogenic effects of SGLT2i would be expected to resemble anti-inflammatory therapies more than antithrombotic agents in their time course. That is, such effects are likely incremental, phenotype-dependent, and slow to influence ischemic event rates, particularly in contemporary post-MI populations already receiving potent antithrombotic and lipid-lowering therapy.

This framework provides a coherent explanation for the neutral results of post-AMI trials of SGLT2i with respect to ischemic coronary MACEs, despite consistent signals on HF-related outcomes. In the early post-infarction phase, clinical events are dominated by thrombotic mechanisms that are rapidly and effectively suppressed by antiplatelet therapy, while plaque-stabilizing and anti-inflammatory effects—whether from statins, colchicine, or potentially SGLT2is—require longer exposure to manifest clinically. Expecting SGLT2i to reduce early recurrent myocardial infarction in a manner analogous to antithrombotic therapy therefore represents a mismatch between mechanism and endpoint. Rather, SGLT2i should be interpreted as agents that favorably shift the post-AMI trajectory toward reduced heart failure and adverse remodeling early, with any anti-atherogenic contribution—if clinically relevant—likely to emerge only over extended follow-up and in patients with substantial residual metabolic and inflammatory risk.

## 6. Conclusions

SGLT2i have transformed cardiovascular therapeutics by consistently reducing HF morbidity and favorably influencing cardiac remodeling across a broad range of clinical settings. In the context of AMI, however, randomized trials have demonstrated a clear dissociation between robust benefits on HF-related outcomes and neutral effects on recurrent ischemic “coronary” events. Rather than representing a failure of biological efficacy, this pattern may reflect a mismatch between mechanism, timing, and endpoint selection.

Accumulating experimental and translational evidence supports a coherent anti-atherogenic framework for SGLT2 inhibition, encompassing indirect effects on cardiometabolic risk factors and direct modulation of key cellular drivers of atherosclerosis, including endothelial cells, VSMC, macrophages, inflammasome signaling, and the PVAT–coronary artery wall axis. These processes may contribute to plaque inflammation and vulnerability over time, rather than acutely preventing thrombosis in the early post-AMI phase dominated by antithrombotic mechanisms and intensive lipid-lowering therapy.

Future research should focus on longer-term post-MI treatment strategies, patient populations characterized by substantial residual metabolic or inflammatory risk, and imaging-guided endpoints capable of capturing changes in plaque biology and vascular inflammation. In this context, advanced invasive and non-invasive imaging modalities, such as OCT [79] and photon-counting computed tomography angiography (PCCTA) [67], may help characterize plaque vulnerability and better assess the potential vascular effects of SGLT2is. Within this perspective, SGLT2i may ultimately prove to be complementary agents that reshape post-AMI risk trajectories by coupling early HF prevention with potential longer-term stabilization of atherosclerotic disease.

## Data Availability

No new data were created or analyzed in this study. Data sharing is not applicable to this article.

## Abbreviations

The following abbreviations are used in this manuscript:

AMI	Acute Myocardial Infarction
HF	Heart Failure
SGLT2is	Sodium–Glucose Cotransporter 2 Inhibitors
T2D	Type 2 Diabetes Mellitus
ESC	European Society of Cardiology
HFrEF	HF with Reduced Ejection Fraction
HFmEF	HF with Mildly Reduced Ejection Fraction
HFpEF	HF with Preserved Ejection Fraction
ROS	Reactive Oxygen Species
ATP	Adenosine Triphosphate
NLRP3	NOD-like Receptor Family, Pyrin Domain–Containing 3
MACE	Major Adverse Cardiovascular Event
ACS	Acute Coronary Syndrome
STEMI	ST-Elevation Myocardial Infarction
LDL	Low-Density Lipoprotein
PVAT	Perivascular Adipose Tissue
VSMC	Vascular Smooth Muscle Cells
eNOS	Endothelial Nitric Oxide Synthase
CCTA	Coronary Computed Tomography Angiography
OCT	Optical Coherence Tomography
DAPT	Dual-Antiplatelet Therapy
PCSK9is	Proprotein Convertase Subtilisin/Kexin Type 9 Inhibitors
PCCTA	Photon-Counting Computed Tomography Angiography
